# Supplementary material for: Leveraging tuberculosis programs for future pandemic preparedness: A retrospective look on COVID-19
Source: PLOS Glob Public Health. 2024 Sep 5;4(9):e0003454. doi: 10.1371/journal.pgph.0003454 (PMC11376540; doi:10.1371/journal.pgph.0003454)
Supplement: Attachment — Submitted filename: ResponseToReviewers.docx [file pgph.0003454.s001.docx]

Dear reviewers,

Thanks for your time spent reviewing our manuscript. We are very grateful and appreciative of your thoughtful comments and suggestions to improve our manuscript.

We offer the following corrections and clarifications to your comments and questions highlighted below, as well as in the marked-up copy of our manuscript which we are re-submitting.

We hope that you will find these revisions satisfactory and will agree to publish our findings which we believe are highly relevant and important to the field of global health. The main conclusion of our manuscript is rebutting the commonly held notion that countries must choose between health systems strengthening *or* pandemic preparedness – rather our findings show that underlying TB program strength had a significant impact on COVID-19 outcomes, particularly in more fragile country settings.

On behalf of the authors, I thank you sincerely again for your comments and suggestions.

Chris Collins

1. Does this manuscript meet PLOS Global Public Health’s publication criteria? Is the manuscript technically sound, and do the data support the conclusions? The manuscript must describe methodologically and ethically rigorous research with conclusions that are appropriately drawn based on the data presented.

Reviewer #1: Partly

Reviewer #2: Yes

We believe that the manuscript is technically sound, with a study design (retrospective observational) appropriate for the questions posed. The data sources utilized are robust and credible, and the statistical analyses performed are suitable for the nature of the data. Given the study utilizes publicly available data that has been anonymized and is non-invasive in nature, this study does not require ethics approval. Conclusions are drawn from multiple quantitative data analyses across various sources and are extremely statistically significant at a threshold significance level of 0.05. Potential limitations to the study are addressed.

2. Has the statistical analysis been performed appropriately and rigorously?

Reviewer #1: I don’t know

Reviewer #2: No

Changes to data analysis are described in more detail below. We believe that with these edits, the statistical analysis has been performed appropriately and rigorously.

3. Have the authors made all data underlying the findings in their manuscript fully available (please refer to the Data Availability Statement at the start of the manuscript PDF file)?

The PLOS Data policy requires authors to make all data underlying the findings described in their manuscript fully available without restriction, with rare exception. The data should be provided as part of the manuscript or its supporting information, or deposited to a public repository. For example, in addition to summary statistics, the data points behind means, medians and variance measures should be available. If there are restrictions on publicly sharing data—e.g. participant privacy or use of data from a third party—those must be specified.

Reviewer #1: No

Reviewer #2: Yes

We have provided all of the data sets and sources that were used in our manuscript. All underlying data was derived from publicly available data from the Global Fund, the World Health Organization, Our World in Data, Github, the World Bank, and National Health Ministries. What came from where is described in detail in the methods section.

4. Is the manuscript presented in an intelligible fashion and written in standard English?

PLOS Global Public Health does not copyedit accepted manuscripts, so the language in submitted articles must be clear, correct, and unambiguous. Any typographical or grammatical errors should be corrected at revision, so please note any specific errors here.

Reviewer #1: Yes

Reviewer #2: Yes

5. Review Comments to the Author

Please use the space provided to explain your answers to the questions above. You may also include additional comments for the author, including concerns about dual publication, research ethics, or publication ethics. (Please upload your review as an attachment if it exceeds 20,000 characters)

**Reviewer #1:**

Comment : It would be good to reorganise the text in the introduction section to include the context of TB before COVID-19. This would help readers to recognise that the focus of this study is about TB control efforts as influenced by the COVID-19 pandemic.

Thanks for this suggestion – we have reworked the introduction to first introduce TB and then pivot to COVID.

Comment: In section I of the Methods section, some equations are not displayed in the downloaded PDF file such as the part about the categories of ‘positive/neutral’ or ‘negative’ recovery for TB.

We’ve inserted equations in a different format that should now download properly.

In section V of the Methods section on case studies, please provide more details about the process of conducting the semi-structured interviews such as sample size and participant recruitment process. Please describe how these results from the case studies would be used alongside the other methods that are focused on quantitative work. Please state the details of the ethics approval. Please include relevant methodological details such as explaining the coding process and saturation. Also, suggest including a summary of topics or questions asked. It would be good to include details about the process of analysing the data.

We have completely reworked the description of the case study methods. We’ve addressed the process of conducting the case interviews, included the sample size, made a statement about the ethics approval, described the coding process/saturation/incorporation with quantitative data, and spoke to how we analyzed the resulting data. As for how interviewees were recruited, this was less of a formal process and more based on contacts we were able to make throughout our research process.

Please include relevant quotes from the interviewees in the results section. It is unclear to me which parts of the sentences are attributed to desk review or semi-structure interviews. These are considered qualitative data that should be included to offer a comprehensive analysis.

We’ve specified this.

Please improve the discussion regarding the strengths and limitations of this study.

Added a comprehensive strengths and limitations section to the manuscript.

Overall, I encourage the authors to reflect on why these findings matter in epidemic and pandemic research

**Reviewer #2:**

Abstract - The sentence “Looking at data from the..” appears incomplete. Should the period be replaced with comma?

Thank you – we’ve fixed this.

Methods – The Data collection and sources of data are very well explained but please specify the study design in the start of the section. Provide more details.

We’ve edited to be more specific.

Line 148 to 151 – Is there a text or figure that needs to appear here?

There was an issue with the equation loading – we’ve edited this.

Line 153 – Not sure, if the line 148 was meant to cover this. But please provide context for Delta treatment coverage. Why are the countries categorized using this variable? Is there a reference for this variable of choice?

What was previously line 148 (which wasn’t loading correctly) covers this. We’ve edited the equation and added a sentence about why we chose to use delta treatment coverage.

Figure 1 – This does not correlate with the text/List in Lines 160-162. Why only few countries are chosen in this chart?

For brevity we chose to only highlight the countries with the 5 best and 5 worst recoveries when comparing TB treatment coverage during and before the pandemic.

Line 232 - Section V. Case Studies – Is this the whole study design? Or is it the last phase after the review of data?

We’ve added significantly to the case study methods section.

Line 232 – How were the interviews conducted? In person or virtual? Were the stakeholders compensated? How was this data analysed? Were they coded? Did you use a software for analysis?

Interviews were conducted virtually. They were not compensated. We’ve specified this in our manuscript and added a lot more information about how the data was analyzed.

Line 237 – Variety of TB control organizations? With Organizations names (MC, TBPPM, PIH, etc.). Do you mean these organizations (MC, TBPPM, PIH, etc.). were the “variety” that you interviewed? Or did you partner with these organizations (MC, TBPPM, PIH, etc.) and interview other organizations?

Clarified in manuscript – we interviewed a mix of people from these organizations and on-the-ground contacts who work directly with ministres of health.

Line 232 to 239 – Why is the case study different from the data collection mentioned above? Why were they all not combined together to report the results?

Financial data and COVID case/death data was collected from different sources than the case studies. The case studies were used qualitatively to supplement and validate quantitative findings. Because the methodology was so different from the financial/case data collection, we separated this into its own section for clarity.

Line 245 – Surveyed? Or Reviewed?

We’ve edited this.

Line 245 to 253 – These numbers seem interesting. Given that this “Data treatment coverage” categorization is important for your study, it will be good to represent this data and the variation among contents in a data matrix.

We’ve added this as a table. Thanks for this suggestion.

Line 346 and Line 372 – How are they different from each other? Both talk about the importance of Global Fund. What is the reason for 2 categorizations?

We aren’t completely sure we understand this comment – part of our analysis talks about the importance of Global Fund financing as an amount, and part of it talks about the share of total financing for a country that comes from the Global Fund. Not sure if this was the difference referred to!

Line 398 – The treatment costs mentioned here, does it denote COVID treatment of TB treatment?

This is treatment costs per TB case. We’ve specified that.

Line 414 to 420 – What do these themes mean? Provide more description.

We state the five themes, then go into much more detail about each of these themes below.

Line 414 to 420 – Consider combining these themes with the themes you have identified from the data review to form meta themes and report the results section?

Thanks – we have looked to combine themes a bit more concisely.

Line 422 – Is this the discussion section of the paper?

The Conclusion and Policy Recommendations was our intended discussion section of the paper.

Line 440 – The reference, Padmapriyadarshini et al does not seem to be there in the references list (Line 568)

Fixed! Thanks.

References – Please verify all references. Some of the references mentioned in the text are not on this list. Few references have the PDF names (e.g. Line 581,582,588,etc.) without any further details. Which organization? Available online? How was it accessed? Etc. Please review citation guidelines.

Fixed! Thanks.
